# Oscillatory Dynamics in Infectivity and Death Rates of COVID-19

**DOI:** 10.1128/mSystems.00700-20

**Published:** 2020-08-18

**Authors:** Tomáš Pavlíček, Pavel Rehak, Petr Král

**Affiliations:** a Institute of Evolution, University of Haifa, Mt. Carmel, Israel; b Department of Chemistry, University of Illinois at Chicago, Chicago, Illinois, USA; c Department of Physics, Biopharmaceutical Sciences, and Chemical Engineering, University of Illinois at Chicago, Chicago, Illinois, USA; University of California at San Diego

**Keywords:** SARS-HCoV-2, computer modeling, infectivity

## Abstract

The analysis of systematically collected data for coronavirus disease 2019 (COVID-19) infectivity and death rates has revealed, in many countries around the world, a typical oscillatory pattern with a 7-day (circaseptan) period. Additionally, in some countries, 3.5-day (hemicircaseptan) and 14-day periodicities have also been observed. Interestingly, the 7-day infectivity and death rate oscillations are almost in phase, showing local maxima on Thursdays/Fridays and local minima on Sundays/Mondays. These observations are in stark contrast to a known pattern correlating the death rate with the reduced medical staff in hospitals on the weekends. While we cannot exclude the possibility that a significant portion of the observed oscillations is associated with the reporting of the individual cases, other reasons might contribute at least partly to these data. One possible hypothesis addressing these observations is that they reflect gradually increasing stress with the progressing week, which can trigger the higher death rates on Thursdays/Fridays. Moreover, assuming the weekends provide the likely time for new infections, the maximum number of new cases might fall, again, on Thursdays/Fridays. These observations deserve further study to provide a better understanding of COVID-19 dynamics.

**IMPORTANCE** The infectivity and death rates for COVID-19 have been observed in many countries around the world as well as in the collective data of the whole world. These oscillations show distinct circaseptan periodicity, which could be associated with numerous biological reasons as well as with improper reporting of the data collected. Since very different results are observed in different countries and even continents, such as Sweden (very significant oscillations) or India (almost no oscillations), these data provide a very important message about different conditions under which the disease is spread or is reported, which, in turn, could serve as guidance tools in future epidemics. It is necessary that follow-up studies track the observed differences and fully reliably address their origins.

## Abstract (precis)

The infectivity and death rates for COVID-19 have been observed in many countries around the world as well as in the collective data of the whole world. These oscillations show distinct circaseptan periodicity, which could be associated with numerous biological reasons as well as with improper reporting of the data collected. Since very different results are observed in different countries and even continents, such as Sweden (very significant oscillations) or India (almost no oscillations), these data provide a very important message about different conditions under which the disease is spread or is reported, which, in turn, could serve as guidance tools in future epidemics. It is necessary that follow-up studies track the observed differences and fully reliably address their origins.

## INTRODUCTION

The current worldwide pandemic caused by severe acute respiratory syndrome coronavirus 2 (SARS-CoV-2) has led within several months to millions of infected individuals and hundreds of thousands of fatalities, while the economies of most countries have been largely put on hold. Therefore, it is urgent to understand the dynamics of coronaviruses, since their repeated straying into the human population could provoke an existential crisis. As a recent report warns (1), dramatic interventions by individual governments to locally reduce the effective reproductive number (*R_t_*) by a lockdown might not substantially change the long-term, total number of infections and possible fatalities if human behavior returns to normal before a vaccine is available (2). Moreover, it becomes obvious that the virus is active in every season, so the number of infected people can locally grow until a significant portion of population gains immunity.

The subfamily *Orthocoronavirinae* (3) comprises the following four genera: *Alphacoronavirus* (containing many animal coronaviruses and two human coronaviruses [HCoVs], HCoV-229E and HCoV-NL63), *Betacoronavirus* (containing mouse hepatitis virus [MHV] and the following human viruses: HCoV-OC43, HCoV-HKU1, Middle East respiratory syndrome coronavirus [MERS-CoV], severe acute respiratory syndrome coronavirus [SARS-CoV], and SARS-CoV-2), and *Gammacoronavirus* and *Deltacoronavirus* (containing viruses from cetaceans, birds, and pigs) (3). As of today, at least seven coronaviruses are already circulating in the human population. However, SARS-CoV-2 has a lot of peculiarities, probably caused by a strong and selective binding of its spike protein to human angiotensin-converting enzyme 2 (ACE2) (4). One of the less obvious peculiarities is a periodic appearance of COVID-19 infectivity and death rates with a typical 7-day oscillatory pattern observed in numerous countries (https://www.worldometers.info/coronavirus/).

Chronobiology describes the mechanisms underlying chronomes, structures in time, which are found in individual organisms, in populations, and in the environment (5). About-7-day (circaseptan) periodicity is frequently found among plants and animals, including humans. As examples of circaseptan periodicity, we can mention the following cases that might be triggered by internal or external factors (6):

- growth of the tail and oviposition in springtails (7),
- the occurrence of cardiovascular events in humans (8),
- the rejection of allografts in rats and humans (9),
- postsurgical swelling decrease after maxillofacial surgery in humans (10),
- many meteorological and pollution variables (11), and
- societal habits relating to periods of rest and activity (6).

In contrast to the circadian clock (12), there is no evidence yet of a molecular mechanism causing circaseptan periodicity. Interestingly, circaseptan periodicity in humans might have given rise to the 6-day working week followed by 1 day of rest, observed around the world. In this weekly periodicity, COVID-19 emerged and possibly locked into the natural circaseptan periodicity, as reflected in the observed periodicity in infectivity and death rates. In certain countries, the weekly repeating maxima and minima of COVID-19 infectivity and related death rates are emerging on certain days with a probability close to certainty. It is particularly striking that the periodicities of COVID-19 infectivity and death rates are almost in phase, and the same pattern is separately followed by very different countries, spectrally analyzed for the presence of possible patterns (13, 25).

A recent study using similar methods analyzed the observed oscillations in data from New York City, NY, and Los Angeles, CA (14, 15). Even though very similar oscillations have been observed in these data, further correlation analysis revealed that the oscillations in the number of new cases can be explained, to a large extent, by the daily variation in testing, while the oscillations in mortality in the U.S. data could, to some extent, be an artifact of reporting. For example, several studies reported a delay in the reporting of new infections and deaths (16, 17). Nevertheless, we cannot exclude more-profound reasons behind some of these observations. For example, even later, a study appeared showing that such oscillations might be due to routine activities and that a lifestyle of more-stressful weekdays flanked by less-stressful, relaxing weekends defines the 7-day immune cycles, where the synchronized low immunity levels in the population are responsible for repeated 7-day waves of pathogenic infections such as COVID-19 (18). Therefore, we feel that it is important to consider additional reasons which can contribute to these observations.

(This article was submitted to an online preprint archive [13]).

## RESULTS

A Fourier transform was used to calculate the spectra associated with the dynamics of new cases and deaths per day from COVID-19 and to reveal possible periodicities present in the reported data in different countries. A detailed analysis was done only for a small set of countries from Europe, the Americas, and Asia, as well as for the whole world. We did not analyze the populations in countries which reported nearly zero cases of coronavirus infection or in countries where the reported numbers of newly acquired cases and deaths could be less reliable.

### Europe.

**FIG 1:**
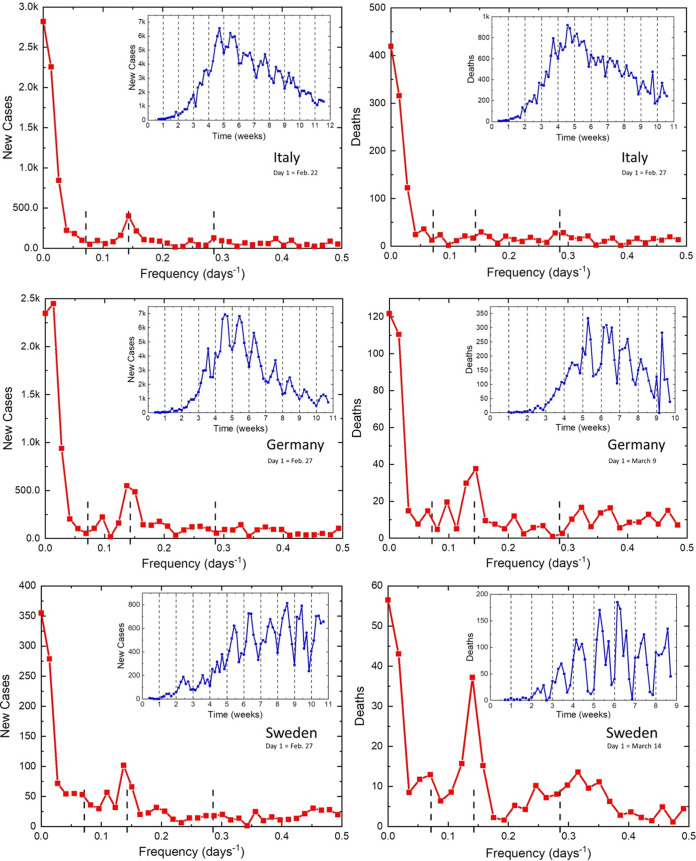
Daily new cases and deaths from COVID-19 reported in Italy (top), Germany (middle), and Sweden (bottom).

Italy was the first European country that was hit very early and severely by COVID-19. It did not have enough time to prepare and react adequately to the severity of the situation and operated under great stress, while gradually strengthening the lockdown rules. Figure 1 reveals strong 7-day and weak 3.5-day periodicities in its infectivity (top left) but much weaker oscillations in the death rate (top right). The minima of infectivity usually took place on Sundays and the minima of the death rate on Mondays, while the maxima were shifted about a half week from the minima.

In contrast, Germany developed COVID-19 infections later and did not implement restrictions for a relatively long time. Figure 1 (middle) reveals very strong oscillations, with a 7-day period present in both parameters studied. These oscillations could reflect the free activity of the virus without limitations caused by a lockdown. An even more relaxed approach was followed in Sweden, which was fully open for a long time. Figure 1 (bottom) reveals the strongest observed oscillations in both parameters, especially the death rate. Here, we can also clearly observe the 14-day and 3.5-day oscillations.

**FIG 2:**
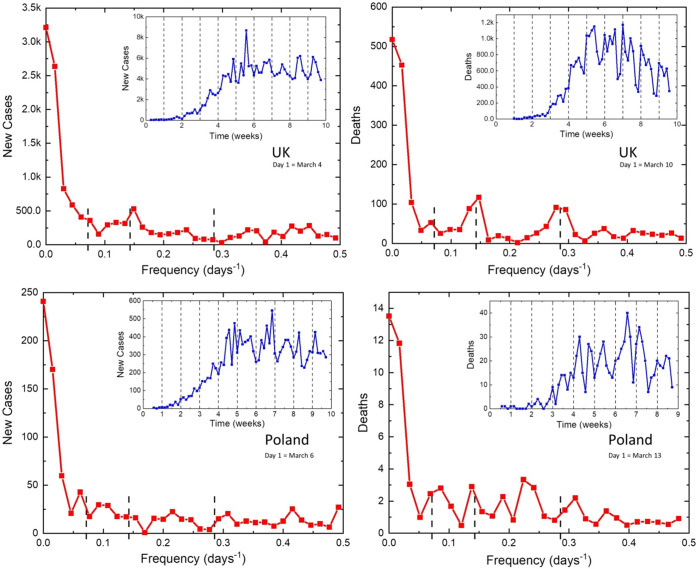
Daily new cases and deaths from COVID-19 reported in the United Kingdom (top) and Poland (bottom).

Other large European countries, such as Spain and France, have also been severely hit by COVID-19, but the periodic oscillations in the observed parameters were not seen clearly enough, possibly due to a more restricted and chaotic development of the situation. Other, smaller countries have also shown medium oscillatory patterns. Among the other large European countries, the United Kingdom (Fig. 2, top) has relatively well-developed 7-day and weak 3.5-day periodicities, perhaps due to relatively relaxed lockdown rules. However, these oscillations were absent in Poland (Fig. 2, bottom).

### Americas.

**FIG 3:**
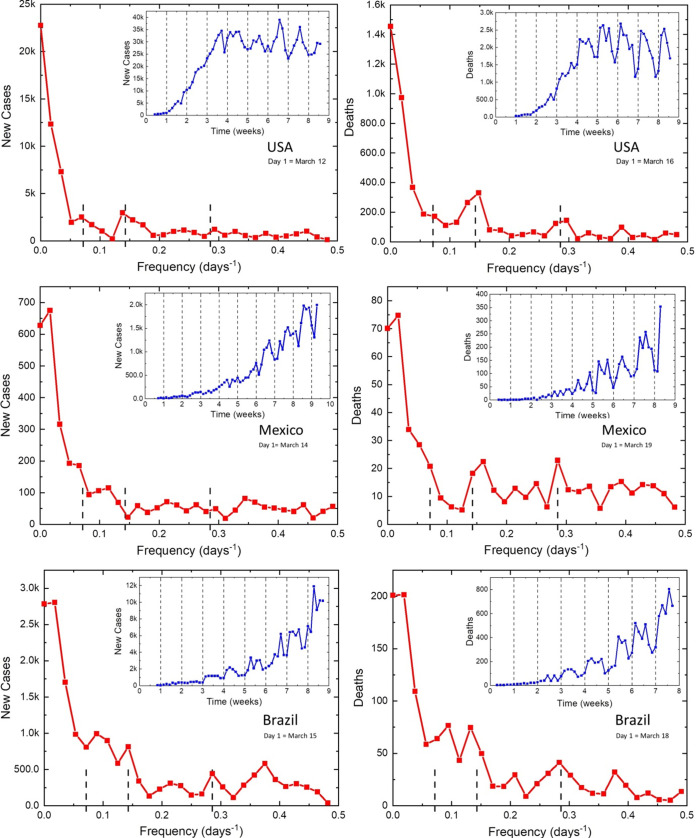
Daily new cases and deaths from COVID-19 reported in the United States (top), Mexico (middle), and Brazil (bottom).

A similar pattern of COVID-19 infection has developed in the Americas. The United States (Fig. 3, top) initially seemed to follow a pattern similar to that of Italy (New York). Later, its oscillatory response turned out to be similar to that of the United Kingdom, where well-developed 7-day and weak 3.5-day periodicities were observed. In contrast, Mexico (Fig. 3, middle) and countries in South America showed relatively strong oscillations, with 14-, 7-, and 3.5-day periods. Interestingly, in Mexico or Peru, the minima are on Sundays to Tuesdays, while in the United States (Fig. 3, top) and Brazil (Fig. 3, bottom), they have a pattern similar to that in Europe.

### Asia.

**FIG 4:**
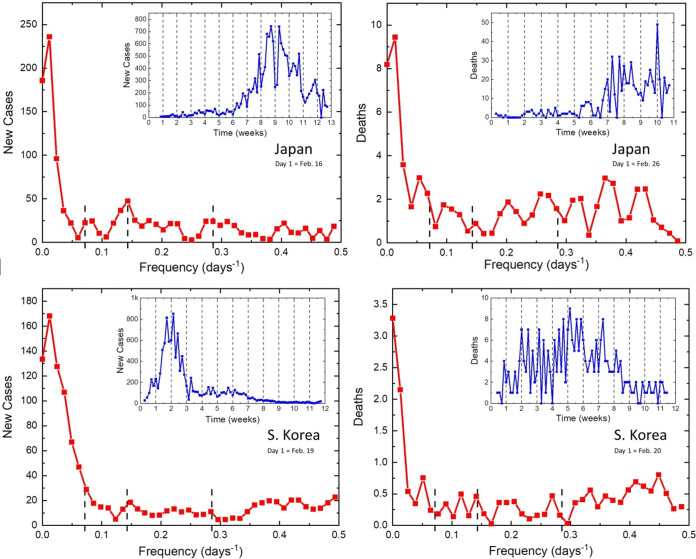
Daily new cases and deaths from COVID-19 reported in Japan (top) and South Korea (bottom).

We have also picked two countries from Asia that provided clear data and were successful in suppressing COVID-19, namely, Japan (Fig. 4, top) and South Korea (Fig. 4, bottom). They both show 7- and 3.5-day periods in infectivity but rather complex patterns in death rates, perhaps affected by the highly controlled nature of the infectivity.

### World.

**FIG 5:**
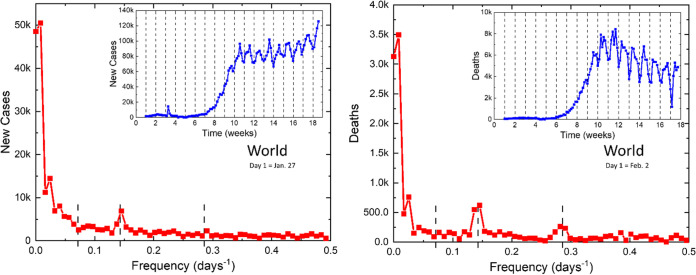
Daily new cases and deaths from COVID-19 reported in the whole world.

Finally, we have provided an analysis in the whole world (Fig. 5), since the collective data became available recently. The results reveal 7- and 3.5-day periods in infectivity and even stronger peaks in death rates. The highly regular oscillations of both parameters observed in the whole world signify the globality of these observations and the relevance of the data obtained. It is worth mentioning that the world oscillations later prevailed throughout the summer. Even though the oscillatory dynamics are clearly visible in many countries, some large countries, such as India, do not reveal it.

## DISCUSSION

Even though we do not know how circaseptan cyclicity is generated in general, we could try to set a hypothesis on the underlying observations for COVID-19, besides the most obvious origin caused by the disparity in measuring and reporting. We can assume that part of these oscillations originates in underlying epigenetic modifications (19) for which SARS-CoV-2 is responsible. This does not exclude the possibility that epigenetic modifications were triggered by changes in meteorological and pollution variables that exhibit a week-long periodicity (11). For example, such variables might be human traffic flow outdoors or poorly working indoor ventilation in hospitals (20). Finally, we cannot exclude the possibility of the desynchronization of the circadian rhythm. For example, it was observed that rectal temperature and urinary temperature desynchronized in a woman staying approximately 3 months in isolation in a subterranean cave from a circatrigintan rhythm (i.e., an oscillation with a frequency of 1 cycle in 30 ± 5 days) (21) and from an internal circasemiseptan rhythm (with a frequency of 1 cycle in 3.5 ± 1 days) (22). Taken together, it seems that the 7-day (± 1-day) periodicity in death minima and maxima contradicts the hypothesis that patients with serious medical conditions are more likely to die in the hospital if they are admitted on a weekend than if they are admitted on a weekday (23). Even though time-dependent variations in stress and immunity can play a certain role in these oscillations (18), a significant portion of the oscillations observed might be associated with the reporting of the individual cases (14–17).

In summary, the circaseptan (7-day ± 1-day) periodicity pattern described, in some cases accompanied by other infradian cycles, such as a hemicircaseptan cycle, is robust in large and reliably reporting countries representing Europe (Italy; Germany, Sweden, United Kingdom), Asia (Japan, South Korea), and North (the United States) and South (Brazil) America, as well as in the whole world. Understanding the relationship between variables associated with the observed periodicities might help in health care improvements, better forecasting of the coronavirus infection, and stock market expectations about economic growth and might help provide a clue for the most suitable times at which expected COVID-19 therapies should be administered (24).

## MATERIALS AND METHODS

The numbers of newly detected cases and death rates from COVID-19 in individual countries are publicly available at the following site: https://www.worldometers.info/coronavirus/. The once-daily updating of the database prevents the detection of circadian (24-h) and ultradian periodicities. Since the time frame of the available data (2020) covers mid-February to mid-May, we could not detect periodicities longer than ∼15 days (e.g., lunar periodicity). Also, the data presented in the above-mentioned database were probably somewhat underestimated. The data underestimations result especially from substantial asymptomatic and presymptomatic transmissions that make containment-based interventions, especially those depending on the recognition of early symptoms or limited testing, more challenging and potentially infeasible (1).

For each country, we recorded (for a finite number of days) the daily number of new cases and deaths. Then, using http://lampx.tugraz.at/~hadley/num/ch3/3.3a.php, we performed a Fourier transform of these dependencies and wrote them as follows:

$$x\left(t\right)=\overset{n<N/2}{\underset{n=0}{\sum }}\left[a_{n}\text{cos}\left(\frac{2\pi \mathrm{nt}}{N\Delta t}\right)+b_{n}\sin\left(\frac{2\pi \mathrm{nt}}{N\Delta t}\right)\right]$$

where *N* is the number of days that we recorded (i.e., the number of data points), Δ*t* is 1 day, $f\left(n\right)=\frac{n}{N\Delta t}$ is a discrete frequency, which is present within the round brackets of the *x(t)* formula and depicted on the horizontal axes of the large graphs, and *a_n_*, *b_n_* are the related Fourier coefficients. From these Fourier coefficients, *a_n_*, *b_n_*, we calculated the power spectrum, *S_xx_*, and its square root, $I(n)=\sqrt{S_{\mathrm{xx}}}=\sqrt{a_{n}^{2}+b_{n}^{2}}$, which was depicted on the vertical axes of the large graphs and further discussed.
